# Serum miR-33a is associated with steatosis and inflammation in patients with non-alcoholic fatty liver disease after liver transplantation

**DOI:** 10.1371/journal.pone.0224820

**Published:** 2019-11-08

**Authors:** Denisa Erhartova, Monika Cahova, Helena Dankova, Marie Heczkova, Irena Mikova, Eva Sticova, Julius Spicak, Ondrej Seda, Pavel Trunecka

**Affiliations:** 1 Department of Hepatogastroenterology, Institute for Clinical and Experimental Medicine, Prague, Czech Republic; 2 Charles University, First Faculty of Medicine, Institute of Physiology, Prague, Czech Republic; 3 Experimental Medicine Centre, Institute for Clinical and Experimental Medicine, Prague, Czech Republic; 4 Clinical and Transplant Pathology Centre, Institute for Clinical and Experimental Medicine, Prague, Czech Republic; 5 Charles University and General University Hospital in Prague, First Faculty of Medicine, Institute of Biology and Medical Genetics, Prague, Czech Republic; Medizinische Fakultat der RWTH Aachen, GERMANY

## Abstract

**Background & aims:**

MiR-33a has emerged as a critical regulator of lipid homeostasis in the liver. Genetic deficiency of miR-33a aggravates liver steatosis in a preclinical model of non-alcoholic fatty liver disease (NAFLD), and relative expression of miR-33a is increased in the livers of patients with non-alcoholic steatohepatitis (NASH). It was unknown whether miR-33a is detectable in the serum of patients with NAFLD. We sought to determine whether circulating miR-33a is associated with histological hepatic steatosis, inflammation, ballooning or fibrosis, and whether it could be used as a serum marker in patients with NAFLD/NASH.

**Methods:**

We analysed circulating miR-33a using quantitative PCR in 116 liver transplant recipients who underwent post-transplant protocol liver biopsy. Regression analysis was used to determine association of serum miR-33a with hepatic steatosis, inflammation, ballooning and fibrosis in liver biopsy.

**Results:**

Liver graft steatosis and inflammation, but not ballooning or fibrosis, were significantly associated with serum miR-33a, dyslipidemia and insulin resistance markers on univariate analysis. Multivariate analysis showed that steatosis was independently associated with serum miR-33a, ALT, glycaemia and waist circumference, whereas inflammation was independently associated with miR-33a, HbA1 and serum triglyceride levels. Receiver operating characteristic analysis showed that exclusion of serum miR-33a from multivariate analysis resulted in non-significant reduction of prediction model accuracy of liver steatosis or inflammation.

**Conclusions:**

Our data indicate that circulating miR-33a is an independent predictor of liver steatosis and inflammation in patients after liver transplantation. Although statistically significant, its contribution to the accuracy of prediction model employing readily available clinical and biochemical variables was limited in our cohort.

## Introduction

NASH has become a major cause of cirrhosis and hepatocellular carcinoma, and represents one of the most common indications for liver transplant in the United States [1]. NASH develops in the context of hepatic steatosis, a process during which hepatocytes accumulate excessive amount of lipids via mechanisms that have only recently been characterized [2] [3]. Liver steatosis develops in up to 50% of liver transplant recipients and shares many etiopathogenetic factors with steatosis in NAFLD patients in general population [4–7].

Recent reports have highlighted the critical role of microRNAs in regulation of hepatic steatosis. MicroRNAs negatively regulate expression of a wide variety of proteins involved in lipid metabolism, and thus post-transcriptionally modify lipolysis, lipogenesis and lipoprotein turnover [8]. In addition, microRNAs are exported from liver cells and their profile in the serum correlates with the underlying mechanism of liver pathology in preclinical models of liver disease [9].

Numerous studies have shown that miR-33 is a critical microRNA regulating metabolism of fatty acids and cholesterol by cooperating with transcription factors SREBP-1 and -2 (sterol-regulatory element-binding protein-1 and -2), respectively, and by regulating expression of genes of lipid and cholesterol synthesis [10] [11]. Mice deficient in miR-33a exposed to high-fat diet develop severe fatty liver disease, compared to wild-type littermates [12]. In a recently published clinical trial, increased expression of miR-33a in the liver was associated with steatohepatitis in morbidly obese humans [13].

Considering the crucial role of miR-33a in hepatic lipid metabolism and its increased presence in steatotic livers in humans, we hypothesized that miR-33a will be increased in the serum of patients with NAFLD and could be used as non-invasive diagnostic marker. We evaluated this hypothesis in a cohort of patients after liver transplant and used this cohort because of its well-defined demography, clinical data, meticulous follow-up and availability of protocol liver biopsy. Here we show that circulating miR-33a is significantly increased in serum of patients with fatty liver disease after liver transplantation, and that circulating miR-33a is independently associated with steatosis and lobular inflammation in liver biopsy.

## Patients and methods

### Patients

One hundred and sixteen liver transplant recipients undergoing protocol liver biopsy during standard post-transplant follow up between May 2015 and May 2017 were enrolled in this prospective study. We excluded patients with known or suspected alcohol abuse after liver transplantation (LTx), with HCV infection of the graft or with corticosteroids administration higher than 5 mg of prednisone per day. The most common indications for LTx in our cohort were biliary disorders (PBC, PSC, overlap PSC/AIH; 33 patients, 28.5%), alcoholic liver disease (28 patients, 24.1%) and HBV, autoimmune hepatitis and cryptogenic cirrhosis (8 patients, 7% each, S1 Fig). Only one patient in our cohort was indicated for LTx because of NASH (0.9%). All patients included in the study signed informed consent for participation in the study. The study conformed to the ethical guidelines of the 1975 Declaration of Helsinki and was approved by the Joint Ethics Committee of the Institute for Clinical and Experimental Medicine and Thomayer Hospital.

### Clinical data and laboratory testing

Blood samples were collected in fasted state in the morning prior to liver biopsy. We measured serum glucose, HbA1c, C-peptide, insulin, triglycerides, total cholesterol, LDL-cholesterol, HDL-cholesterol, bilirubin, ALT, AST, blood count, and creatinine. All these analyses were performed in an accredited biochemistry laboratory according to the standard manufacturer’s protocols. Homeostatic model assessment index (HOMA-IR), the quantitative insulin-sensitivity check index (QUICKI) and the glomerular filtration rate (MDRD-GFR) were calculated using standard formulas. We also analysed clinical and anthropometric data, including age, body mass index (BMI), waist circumference, time from liver transplantation (LTx), presence of comorbidities such as hypertension, diabetes, and prescription medications (especially immunosuppressive drugs). Next, we analysed clinical and anthropometric data of liver graft donors.

### Circulating miRNAs relative expressions analysis

Blood samples for miRNA relative expressions analysis were collected at the same time as blood samples for biochemical testing on same day when liver biopsy was performed. Serum was separated by centrifugation and stored at -80°C. MiRNAs were isolated from serum using miRCURY RNA isolation kit for biofluids (EXIQON). Before isolation, serum samples were spiked with control miRNA cel-miR-39-3p (QIAGEN), which served as control of quality of isolation and as reference gene. RNAcarrier—bacteriofage MS2 (Roche) was added for better yield.

### Liver biopsy

Liver biopsies were performed using the Menghini technique. Histologic sections of formalin-fixed, paraffin-embedded liver tissue were routinely stained according to the standard protocols. All samples were reviewed by an experienced histopathologist who was blinded to miRNA analysis. Liver biopsies were graded according to the scale published by Kleiner, where four morphological features related to NAFLD were semi-quantitatively appraised on light microscopy: steatosis, lobular inflammation, hepatocellular ballooning and fibrosis [14].

### Statistical analysis

One-way ANOVA or Mann-Whitney test, when appropriate, were used for comparison of continuous variables, and chi-square or Fisher’s exact test were used for comparison of proportions. To assess the role of miRNAs, clinical and demographic variables in clinical outcomes, stepwise logistic regression was used. Predicted probability values for clinical outcomes calculated in regression models were used to construct receiver operating characteristic curves. Goodness-of-fit statistics were assessed for all regression models. The level of significance was set at P less than 0.05. All P values were two sided. Statistical calculations were performed using SPSS Statistics Version 25 (IBM Corporation).

## Results

### Cohort characteristics

Demographic and clinical data of all patients enrolled in the study are shown in Table 1 and S1 Table. In total, 116 patients (median BMI was 25.3 kg/m^2^) in stable clinical condition participated in the study (60 men and 56 women). Median time from LTx was 2.2 years (minimum: 1 year, maximum: 20.5 years). Thirty-one patients had diabetes and 70 patients had hypertension. On average, plasma levels of aminotransferases were within normal range (Table 1). Donor characteristics and immunosupresion regimens are described in S1 Table.

**Table 1:** Demographic and clinical characteristics of enrolled patients. Data are given as N (%) or median (1^st^ - 3^rd^ quartile).

	N = 116	
**Male gender**	60 (52%)	
**Age** [years]	56.8 (42.0–64.8)	
**Time from LTx** [days]	805 (446–2381)	
**BMI** [kg/m^2^]	25.3 (22.4–29.5)	
**Waist circumference** [cm]	96 (85–107)	
**Hypertension**	70 (60.3%)	
**Diabetes**	31 (27%)	
**Statins**	19 (16.4%)	
**Laboratory values:**		**Reference range:**
**Bilirubin [**μmol/L**]**	12.2 (9.2–18)	3.4–20
**AST** [μkat/L]	0.40 (0.33–0.47)	0.17–0.75
**ALT** [μkat/L]	0.45 (0.37–0.59)	0.17–1.17
**Glycaemia** [mmol/L]	5.3 (4.9–6.0)	3.6–5.59
**HbA1c** [%]	37.0 (32.8–41.3)	20–42
**C-peptide** [nmol/L]	0.8 (0.6–1.1)	0.26–1.03
**Insulinemia** [mIU/mL]	7.6 (4.8–10.0)	2.1–22
**HOMA-IR**	1.8 (1.1–2.6)	0.5–1.4
**QUICKI**	0.35 (0.33–0.38)	0.45–0.339
**Triglycerides** [mmol/L]	1.1 (0.8–1.6)	0.5–1.69
**Total cholesterol** [mmol/L]	4.5 (3.8–5.0)	2.9–5
**LDL-cholesterol** [mmol/L]	2.6 (2.0–3.1)	1.2–3
**HDL-cholesterol** [mmol/L]	1.2 (1.0–1.4)	1–2.1
**Creatinine [**μmol/L**]**	92 (79–115)	49–90
**MDRD-GFR** [ml/min/1.73^2^]	68 (53–80)	> 80
**WBC** [x10^9^/L]	6.1 (4.9–7.3)	4–10
**Erythrocytes** [x10^12^/L]	4.7 (4.3–5.1)	3.8–5.2
**Haemoglobin** [g/L]	136 (125–150)	120–160
**Thrombocytes** [x10^3^/l]	174 (143–219)	150–400
**CRP** [mg/L]*	2.4 (1–4.5)	0–5

* CRP was measured only in 59 patients from the cohort

**Fig 1:**
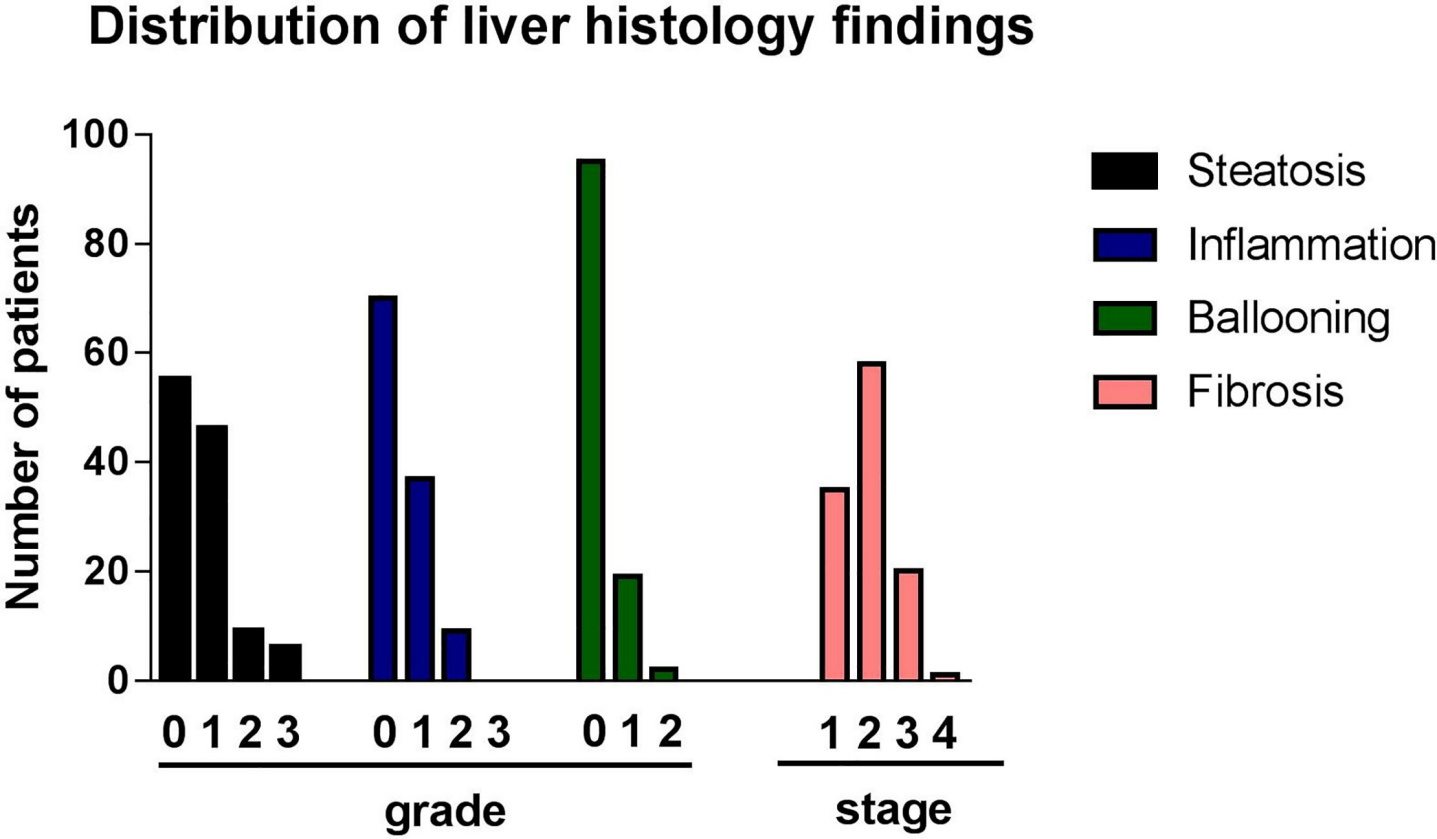
Distribution of liver histology findings in our cohort. Steatosis, lobular inflammation, ballooning of any grade, and fibrosis of any stage were present in 53%, 40%, 18% and 100% patients, respectively. Advanced degree of steatosis (≥2), lobular inflammation (≥2), ballooning (≥2) or fibrosis (≥3) was present in 13%, 8%, 2% and 18% patients, respectively.

Liver biopsy findings are shown in Fig 1. Steatosis, lobular inflammation, ballooning of any grade, and fibrosis of any stage were present in 53%, 40%, 18% and 100% patients, respectively (Fig 1). Advanced degree of steatosis (≥2), lobular inflammation (≥2), ballooning (≥2) or fibrosis (≥3) was present in 13%, 8%, 2% and 18% patients, respectively (Fig 1).

### Circulating miR-33a is increased in patients with liver graft steatosis and lobular inflammation

Given its crucial role in lipid metabolism regulation, we compared serum levels of miR-33a in patients with no steatosis and patients with liver graft steatosis (Table 2). In order to validate this analysis, we also evaluated serum levels of two miRNAs with known association with NASH (miR-34a and miR-122, positive validation) and one miRNA not previously associated with NASH (miR-106b, negative validation). Next, we compared serum of those four miRNAs in patients with and without lobular inflammation, with and without ballooning and with and without fibrosis.

**Table 2:** Associations of miRNAs with liver biopsy findings. Top row shows fold changes and 95% confidence intervals. Bottom row shows p-values derived from 1-way ANOVA. Statistically significant results are printed in bold.

	Steatosis	Inflammation	Ballooning	Fibrosis
**miR-33a**	**1.17 (1.04–1.30)** **0.034**	**1.25 (1.08–1.42)** **0.003**	1.10 (0.86–1.35) 0.30	1.02 (0.93–1.11) 0.79
**miR-34a**	1.42 (0.74–2.10) 0.28	**1.96 (0.88–3.04)** **0.030**	2.23 (0.27–4.20) 0.86	1.38 (0.81–1.96) 0.39
**miR-122**	1.19 (0.92–1.46) 0.29	**1.46 (1.06–1.87)** **0.031**	**1.57 (1.02–2.12)** **0.023**	0.76 (0.60–0.92) 0.13
**miR-106b**	1.06 (0.73–1.38) 0.62	1.04 (0.74–1.34) 0.83	1.18 (0.57–1.80) 0.36	1.05 (0.79–1.36) 0.82

We found that serum miR-33a was associated with liver graft steatosis and lobular inflammation. Specifically, patients with steatosis on liver biopsy had 17% (95% CI 1.04–1.30) increase of serum miR-33a, compared to subjects without steatosis (*p* = 0.034, Table 2), and patients with lobular inflammation on liver biopsy had 25% (95% CI 1.08–1.42) increase of serum miR-33a in comparison to subjects without inflammation (*p* = 0.003, Table 2). There was no association between miR-33a and ballooning or fibrosis (Table 2). Consistent with published reports, miR-34a and miR-122, used for positive validation, but not miR-106, used for negative validation, showed a significant increase in patients with liver inflammation [15].

### Circulating miR-33a is an independent predictor of graft steatosis in context of clinical and laboratory characteristics

The increased serum levels of miR-33a in patients with steatosis prompted us to investigate whether miR-33a has a potential role as non-invasive biomarker of graft steatosis. We also postulated that if such role exists, miR-33a should be associated with graft steatosis independently of demographic, clinical, and biochemical variables.

To answer this question, we first used univariate analysis to identify demographic, clinical and biochemical variables associated with graft steatosis. To do so we divided all patients into group without steatosis and group with steatosis (Table 3). This analysis showed that in addition to serum miR-33a, steatosis was also associated with laboratory or demographic variables including age, BMI, waist circumference, diabetes, treatment with statins, ALT, glycaemia, HbA1c, C-peptide, HOMA-IR and triglycerides. Multivariate regression analysis showed that miR-33a was independent predictor of graft steatosis. For each fold-change of miR-33a serum level, the odds ratio for graft steatosis increased 2.9-fold (Table 4). In addition, glycaemia, waist circumference and ALT were found to be independent predictors of graft steatosis as well, consistently with previous reports [4].

**Table 3:** Steatosis. **Univariate analysis of the effect of clinical and laboratory findings on developing graft steatosis.** Data are given as N (%) or median (1^st^ - 3^rd^ quartile). Significant results are printed in bold. Normal ranges of biochemical values are mentioned in Table 1. Non-steatosis group includes subjects without histologically proven graft steatosis (≤ 5% of hepatocytes); steatosis group comprises all patients with steatosis grade 1–3.

	Non-steatosis N = 55 (47.4%)	Steatosis N = 61 (52.6%)	p-value
**Male gender**	23 (41.8%)	37 (60.7%)	0.06
**Age** [years]	**48.7 (37.5–59.2)**	**60.1 (50.8–65.9)**	**0.001**
**Time from LTx** [days]	798 (471–3711)	894 (445–1916)	0.51
**BMI** [kg/m^2^]	**23.6 (21.5–27.4)**	**27.3 (24.5–31.1)**	**< 0.001**
**Waist circumference** [cm]	**88 (81.5–96)**	**103 (92–111)**	**< 0.001**
**Hypertension**	29 (52.7%)	41 (67.2%)	0.13
**Diabetes**	**8 (14.5%)**	**23 (37.7%)**	**0.006**
**Statins**	**5 (9.1%)**	**14 (23%)**	**0.049**
**Liver function tests:**			
**Bilirubin [**μmol/L**]**	12.1 (9.4–19.2)	12.4 (9.1–17.1)	0.67
**AST** [μkat/L]	0.39 (0.32–0.45)	0.40 (0.34–0.50)	0.12
**ALT** [μkat/L]	**0.41 (0.34–0.49)**	**0.52 (0.40–0.69)**	**0.021**
**Glucose metabolism:**			
**Glycaemia** [mmol/L]	**5.2 (4.9–5.6)**	**5.5 (4.9–6.5)**	**0.003**
**HbA1c** [%]	**36 (32–39)**	**38 (33–45)**	**0.010**
**C-peptide** [nmol/L]	**0.7 (0.6–0.9)**	**0.8 (0.6–1.2)**	**0.030**
**Insulinemia** [mIU/mL]	7.2 (4.6–9.1)	7.6 (5.0–11.2)	0.06
**HOMA-IR**	**1.7 (1.0–2.2)**	**2.1 (1.1–3.2)**	**0.01**
**QUICKI**	0.35 (0.34–0.38)	0.34 (0.32–0.38)	0.19
**Lipid metabolism:**			
**Triglycerides** [mmol/L]	**1.0 (0.8–1.3)**	**1.2 (0.9–1.9)**	**0.004**
**Total cholesterol** [mmol/L]	4.4 (3.8–4.9)	4.5 (3.8–5.2)	0.27
**LDL-cholesterol** [mmol/L]	2.5 (2.1–3.0)	2.6 (1.9–3.2)	0.42
**HDL-cholesterol** [mmol/L]	1.3 (1.0–1.5)	1.1 (0.9–1.4)	0.07
**Renal function:**			
**Creatinine [**μmol/L**]**	91 (76–101)	99 (82–121)	0.08
**MDRD-GFR** [ml/min/1.73^2^]	73 (54–82)	63 (51–77)	0.11
**Blood count:**			
**WBC** [x10^9^/L]	5.9 (4.8–7.3)	6.2 (5.1–7.5)	0.86
**Erythrocytes** [x10^12^/L]	4.6 (4.3–5.0)	4.8 (4.3–5.1)	0.39
**Haemoglobin** [g/L]	136 (122–148)	136 (128–152)	0.39
**Thrombocytes** [x10^3^/L]	184 (142–238)	172 (145–216)	0.34
**CRP** [mg/L]*	1.8 (0.8–3.1)	2.9 (1.3–5.7)	0.06
**miRNAs:**			
**miR-33a**	**1.19 (0.96–1.48)**	**1.29 (0.89–1.76)**	**0.034**
**miR-34a**	1.37 (0.91–2.65)	1.76 (1.15–3.02)	0.28
**miR-106b**	1.62 (1.06–2.18)	1.37 (1.03–2.12)	0.62
**miR-122**	3.31 (1.44–5.95)	3.99 (2.05–8.44)	0.29

* CRP was measured only in 59 patients from the cohort.

**Table 4:** Steatosis. **Multivariate logistic regression involving all significant variables from univariate analysis (including miR-33a).** ALT underwent logarithmic transformation.

	p-value	Odds ratio	95% CI	Wald
**miR-33a**	0.039	2.86	1.06–7.75	4.27
**waist circumference**	< 0.001	1.07	1.03–1.11	13.63
**glycemia**	0.034	1.51	1.03–2.21	4.48
**ALT**	0.026	3.71	1.17–11.79	4.93

### Circulating miR-33a is an independent predictor of liver graft inflammation in context of clinical and laboratory characteristics

Next, we asked whether miR-33a is independently associated with liver graft inflammation. Hence, we divided all patients into two groups. The first one included patients without lobular inflammation in their liver biopsy and the second one included patients with present lobular inflammation. Based on the results of univariate analysis, we analysed miR-33a along with age, BMI, waist circumference, diabetes, glycaemia, HbA1c, C-peptide, HOMA-IR, triglycerides, total cholesterol plasma concentration, glomerular filtration rate (MDRD-GFR) and C-reactive protein (CRP) (Table 5). In addition, we found that liver graft inflammation was also associated with miR-34a and miR-122 in univariate analysis (Table 2 and Table 5). Multivariate regression analysis showed that miR-33a, but not miR-34a or miR-122 was an independent predictor of lobular inflammation. For each fold-change of miR-33a serum level, the odds ratio for lobular inflammation was increased 4-fold (Table 6). In addition, HbA1c and triglycerides were found to be independent predictors of lobular inflammation as well.

**Table 5:** Lobular inflammation. **Univariate analysis of the effect of clinical and laboratory findings on developing liver graft inflammation.** Data are given as N (%) or median (1^st^ - 3^rd^ quartile). Significant results are printed in bold. Normal ranges of biochemical values are mentioned in Table 1. Non-lobular inflammation group includes subjects without histologically proven lobular inflammation; lobular inflammation group comprises all patients with lobular inflammation grade 1–3.

	Non- inflammation N = 70 (60.3%)	Lobular inflammation N = 46 (39.7%)	p-value
**Male gender**	35 (50%)	25 (54.3%)	0.71
**Age** [years]	**52.5 (40.9–62.4)**	**60.1 (50.8–65.3)**	**0.038**
**Time from LTx** [days]	898 (482–3680)	771 (433–1901)	0.39
**BMI** [kg/m^2^]	**24.2 (21.9–28.2)**	**27.7 (24.5–30.9)**	**0.002**
**Waist circumference** [cm]	**91 (83–103)**	**103 (93–113)**	**< 0.001**
**Hypertension**	40 (57.1%)	30 (65.2%)	0.44
**Diabetes**	**11 (15.7%)**	**20 (43.5%)**	**0.001**
**Statins**	10 (14.3%)	9 (19.6%)	0.46
**Liver function tests:**			
**Bilirubin [**μmol/L**]**	12.4 (9.4–19.3)	12 (9.1–16.3)	0.42
**AST** [μkat/L]	0.40 (0.32–0.45)	0.39 (0.34–0.54)	0.15
**ALT** [μkat/L]	0.44 (0.36–0.52)	0.53 (0.39–0.70)	0.09
**Glucose metabolism:**			
**Glycaemia** [mmol/L]	**5.2 (4.9–5.7)**	**5.6 (5.0–6.8)**	**0.003**
**HbA1c** [%]	**36 (32–40)**	**39 (34–47)**	**< 0.001**
**C-peptide** [nmol/L]	**0.7 (0.5–0.9)**	**0.8 (0.7–1.1)**	**0.032**
**Insulinemia** [mIU/mL]	7.1 (5.0–9.5)	8.1 (4.3–11.4)	0.13
**HOMA-IR**	**1.6 (1.1–2.5)**	**2.2 (1.0–3.2)**	**0.037**
**QUICKI**	0.36 (0.33–0.38)	0.34 (0.32–0.38)	0.21
**Lipid metabolism:**			
**Triglycerides** [mmol/L]	**1.0 (0.8–1.3)**	**1.3 (1.0–2.1)**	**< 0.001**
**Total cholesterol** [mmol/L]	**4.2 (3.7–4.9)**	**4.7 (4.1–5.3)**	**0.019**
**LDL-cholesterol** [mmol/L]	2.4 (1.9–3.0)	2.7 (2.3–3.2)	0.06
**HDL-cholesterol** [mmol/L]	1.3 (1.0–1.5)	1.1 (0.9–1.4)	0.08
**Renal function:**			
**Creatinine [**μmol/L**]**	91 (77–104)	100 (83–123)	0.07
**MDRD-GFR** [ml/min/1.73^2^]	**73 (54–83)**	**63 (50–76)**	**0.032**
**Blood count:**			
**WBC** [x10^9^/L]	5.8 (4.8–7.2)	6.6 (5.5–7.9)	0.08
**Erythrocytes** [x10^12^/L]	4.7 (4.4–5.0)	4.8 (4.3–5.2)	0.40
**Haemoglobin** [g/L]	135 (125–148)	142 (125–151)	0.56
**Thrombocytes** [x10^3^/L]	173 (140–218)	176 (146–223)	0.60
**CRP** [mg/L]*	**1.8 (0.75–3.4)**	**3.1 (1.4–5.2)**	**0.027**
**miRNAs:**			
**miR-33a**	**1.18 (0.92–1.50)**	**1.35 (0.93–1.92)**	**0.003**
**miR-34a**	**1.37 (0.94–2.39)**	**2.09 (1.16–3.22)**	**0.03**
**miR-106b**	1.41 (1.06–2.06)	1.38 (1.01–2.41)	0.83
**miR-122**	**3.34 (1.46–5.61)**	**4.97 (1.98–10.14)**	**0.031**

* CRP was measured only in 59 patients from the cohort.

**Table 6:** Lobular inflammation. Multivariate logistic regression involving all significant variables from univariate analysis (including miR-33a, miR-34a, miR-122).

	p-value	Odds ratio	95% CI	Wald
**miR-33a**	0.006	3.95	1.49–10.46	7.62
**HbA1c**	0.049	1.07	1.00–1.15	3.87
**Triglycerides**	0.053	2.40	0.99–5.82	3.74

### Clinical utility of miR-33a in non-invasive diagnosis of liver graft steatosis or inflammation

**Fig 2:**
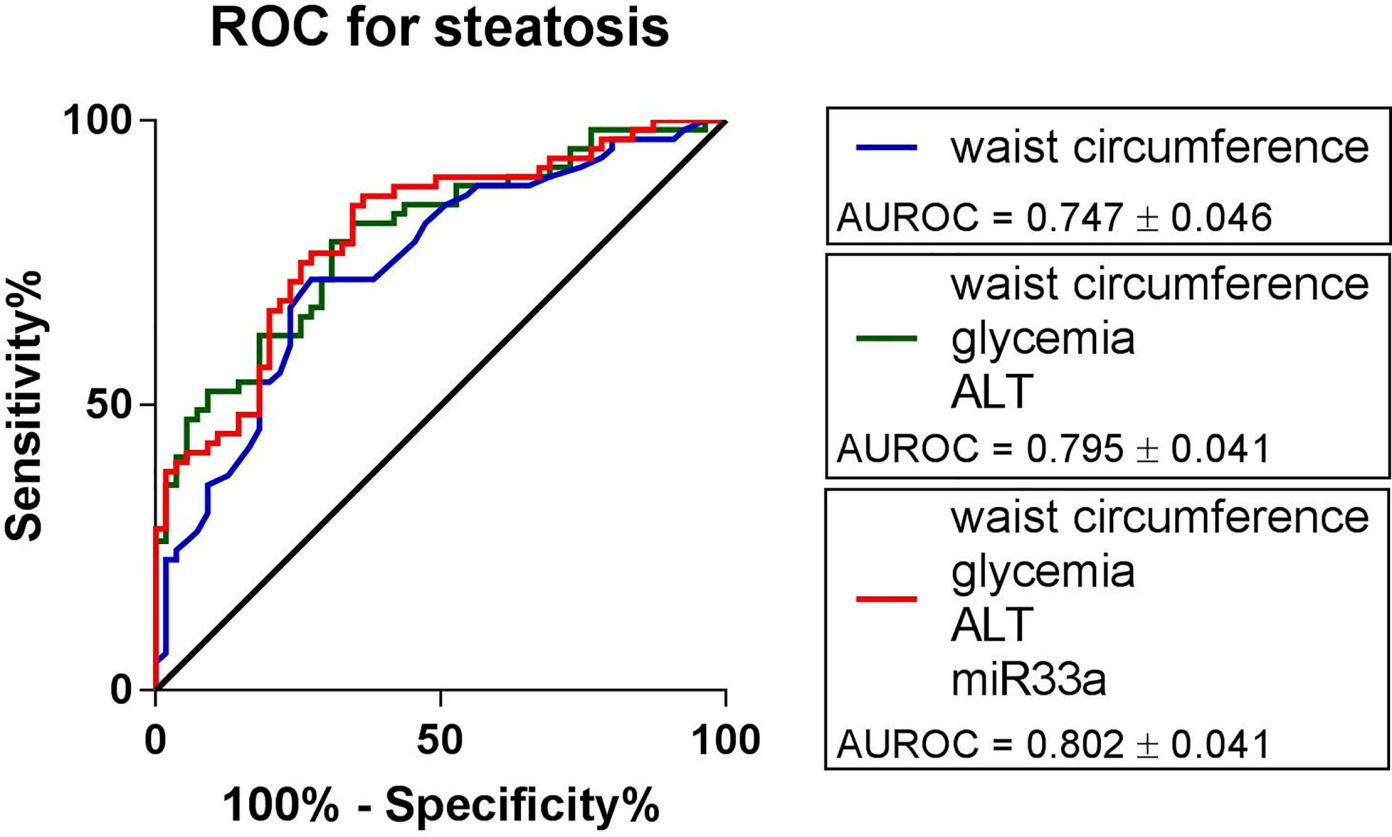
ROC curve for liver graft steatosis. ROC curve for steatosis shows that exclusion of miR-33a from the model decreased the accuracy of the regression model by 0.7%.

Multivariate regression showed that miR-33a, glycaemia, waist circumference and ALT represent independent predictors of hepatic steatosis. Based on the Wald’s statistics, which assesses the relative contribution of each variable to the outcome in regression model, we hypothesized that the relative significance of miR-33a (Wald’s coefficient 4.3) in prediction of liver graft steatosis will be, at most, at par with clinical and laboratory parameters (waist circumference (Wald’s coefficient 13.6), glycaemia (Wald’s coefficient 4.5), ALT (Wald’s coefficient 4.93)) (Table 4). To confirm or reject this hypothesis, we constructed a receiver operating characteristics (ROC) curve, which showed that inclusion of all four variables rendered 80.2% accuracy of the regression model for steatosis (Fig 2). Exclusion of miR-33a from the model decreased the accuracy of the regression model by 0.7% to 79.5% (Fig 2).

**Fig 3:**
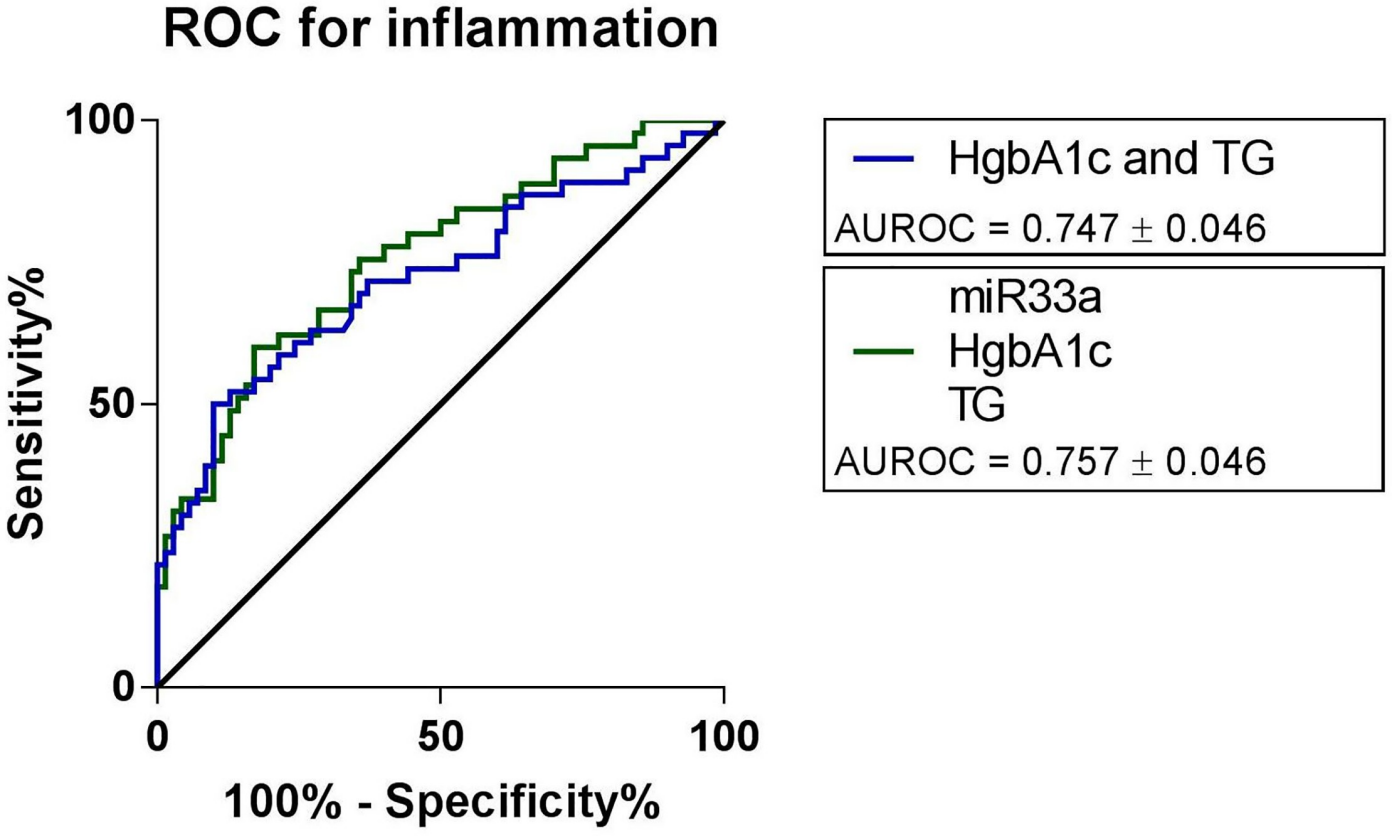
ROC curve for lobular inflammation. ROC curve for liver graft inflammation shows that exclusion of miR-33a from the model decreased the accuracy of the regression model by 1%.

Utilizing a similar approach for assessment of relative contribution of miR-33a to lobular inflammation in regression model, we found that the area under the curve (AUROC) for all three independent predictors (serum miR-33a, triglycerides, HbA1c) was 75.7%, whereas exclusion of miR-33a decreased accuracy to 74.7% (Fig 3). Taken together, although serum miR-33a was an independent predictor of liver graft steatosis or lobular inflammation, its contribution to the predictive model was limited.

## Discussion

Our results have shown that serum levels of miR-33a are significantly increased in liver transplant recipients with graft steatosis or lobular inflammation. Using multivariate regression analysis we showed that miR-33a is an independent predictor of liver graft steatosis or lobular inflammation in the context of clinical and biochemical variables. To the best of our knowledge this is the first study that described increased circulating miR-33a in patients with NAFLD, and these novel findings are consistent with our current understanding of the role of miR-33a in lipid metabolism. We believe, that as the transplant recipients with graft steatosis share most of risk factors associated with NAFLD in general population, particularly high prevalence of risk alleles of *PNPLA3* rs738409 and *TM6SF2* rs58542926 gene polymorphism in corresponding donors, higher BMI, higher triglycerides plasma concentration and diabetes mellitus [4,16,17], our finding could probably, with caution, also apply to regular NAFLD/NASH patients.

Our finding of increased serum level of miR-33a in patients with liver graft steatosis is consistent with the known role of miR-33a in lipid metabolism. First, miR-33 is encoded by an intronic sequence within genetic loci encoding SREBP-1 and SREBP-2, two transcription factors critically involved in regulation of fatty acid and cholesterol homeostasis [10] [11]. Second, expression of miR-33, along with expression of SREBP-1 and -2, is upregulated by insulin resistance, which has a causal role in pathogenesis of NAFLD [18]. Third, suppression of miR-33a by genetic approaches or by therapeutic RNA in preclinical models of NAFLD [12] [19] resulted in the development of liver steatosis or in major changes of plasma lipoprotein profile. Although our findings suggest that the increased serum levels of miR-33 may reflect increased expression of SREBP-1 and -2 driving an increased lipid and cholesterol synthesis, we cannot completely rule out the possibility that increased miR-33 reflects insulin resistance rather than increased lipogenesis. Similarly, we cannot attribute the increased levels of miR-33 in the serum solely to its release from liver cells as miR-33 (and SREBP-1) are expressed in all tissues metabolizing lipids or cholesterol, albeit to a lesser degree compared to hepatocytes [11].

Although we identified miR-33a as independent predictor of liver graft steatosis and inflammation, it needs to be emphasized that statistical significance does not always imply clinical relevance. Using ROC analysis we showed only limited contribution of miR-33a to prediction modelling of steatosis and lobular inflammation in the context of other independent clinical or demographic predictors. This finding of limited clinical relevance is consistent with previously published reports, that showed that AUROC of microRNA (namely miR-34a miR-122) in the diagnostics of lipid accumulation or liver inflammation was between 0.6–0.8 [20]. Not only miRNAs, that failed to act as useful biomarker of NAFLD/NASH but most of other recently suggested noninvasive biomarkers are lacking sufficient discriminatory power, or posses other shortcommings preventing them from use in routine clinical practice [21].

The inherent drawback of using miRNAs as non-invasive markers of liver disease relies in their mechanism of activation, which is usually dependent upon gene or metabolic pathways they are part of. If, as it is often in the case of NAFLD, those genes are involved in glucose or lipid regulation, then readily available laboratory markers of insulin resistance or dyslipidemia will provide similar diagnostic information and therefore it would be of no surprise that adding the corresponding miRNA does not further contribute to the diagnostic model accuracy.

We used a cohort of liver transplant recipients for our study because we believe that these represent appropriate in vivo model of liver steatosis and steatohepatitis demonstrating most of the epidemiologic and genetic risks described in general population [5,16,17]. We are not aware of any difference between pathogenetic mechanisms employed in NAFLD in general population and in liver transplant recipients. It was also described previously that graft steatosis is not transferred from the donor as it rather resolves shortly after transplantation [22–24]. Despite we have found no association between immunosuppressive treatment and development of graft steatosis after liver transplantation in cohort of 268 liver transplant recipients, this influence cannot be easily ruled out [17]. The advantage of this cohort is that graft steatosis after liver transplant develops with high prevalence (20–40% on average) and faster than in general population [5] [6]. In addition, liver transplant recipients have close follow-ups including protocol liver biopsy, which is still the gold standard in diagnosis of NAFLD. We also used the two most investigated miRNAs (miR-34a and miR-122) in non-transplanted patients with NAFLD as positive validation of our results and they were also upregulated in case of liver transplant recipients. Next, we are aware that most of our patients had mild NAFLD phenotype, and we believe that including patients with more advanced NAFLD could unravel greater contribution of serum miR-33a to the liver phenotypes investigated in this study.

Nevertheless it is necessary to keep in mind that transplanted patients are specific cohort, due to immunosuppression including steroids, (described in detail in S1, S2 and S3 Tables), and host-graft interactions which are nowadays not completely understood. Taken all together, patients with NAFLD after liver transplantation has many similarities with the general NAFLD population and probably serve as valuable model, but the findings should apply to general population with considerable caution.

In conclusion, we have shown that circulating miR-33a is associated with steatosis and inflammation in patients with non-alcoholic fatty liver disease after liver transplantation. If validated in more robust cohorts of patients with more advanced stages of NAFLD/NASH, preferably from general population, miR-33a could potentially be used as a useful biomarker.

## Supporting information

S1 Table Donor characteristics and immunosupresion regimens—All patients enrolled in study. Data are given as N (%) or median (1^st^ - 3^rd^ quartile).
(DOCX)

S2 Table Donor characteristics and immunosupresion regimens—Steatosis. Data are given as N (%) or median (1^st^ - 3^rd^ quartile).
(DOCX)

S3 Table Donor characteristics and immunosupresion regimens—Lobular inflammation. Data are given as N (%) or median (1^st^ - 3^rd^ quartile).
(DOCX)

S1 Fig Graph of indications for liver transplant in our cohort. Biliary cirrhosis was indication for LTx in 33 patients (28.5%), alcoholic liver disease in 28 (24.1%), HBV, autoimmune and cryptogenic in 8 patients each (7%), HCV in 4 patients (3.4%), NASH in 1 patient (0.9%) and other diagnoses in 26 patients (22.4%).
(JPG)

S1 Data Underlying data of all patients enrolled in the study. (XLSX)

## Abbreviations

AIH: autoimmune hepatitis
ALT: alanine aminotransferase
AST: aspartate aminotransferase
AUROC: area under the receiver operating characteristic curve
BMI: body mass index
Hb1Ac: haemoglobin A1c
HBV: hepatitis B virus
HCV: hepatitis C virus
HDL-cholesterol: high-density lipoprotein cholesterol
HOMA-IR: homeostatic model assessment of insulin resistance
LDL-cholesterol: low-density lipoprotein cholesterol
LTx: liver transplant
MDRD-GFR: modification of diet in renal disease–glomerular filtration rate
miRNA: micro ribonucleic acid
NAFLD: non-alcoholic fatty liver disease
NASH: non-alcoholic steatohepatitis
PCR: polymerase chain reaction
QUICKI: quantitative insulin sensitivity check index
RNA: ribonucleic acid
ROC: receiver operating characteristics
SPSS: statistical package for the social sciences
SREBP: sterol regulatory element-binding protein
PBC: primary biliary cholangitis
PSC: primary sclerosing cholangitis
