# Supplementary material for: Serum miR-33a is associated with steatosis and inflammation in patients with non-alcoholic fatty liver disease after liver transplantation
Source: PLoS One. 2019 Nov 8;14(11):e0224820. doi: 10.1371/journal.pone.0224820 (PMC6839850; doi:10.1371/journal.pone.0224820)
Supplement: S1 Table — Data are given as N (%) or median (1st - 3rd quartile). (DOCX) [file pone.0224820.s001.docx]

|  | **N = 116** |
| --- | --- |
| **Immunosupresion:** |  |
| **Tacrolimus** | 104 (89.7%) |
| **Cyclosporine** | 11 (9.5%) |
| **Mycophenolate mofetil** | 73 (62.9%) |
| **Azathioprine** | 2 (1.7%) |
| **Corticosteroids** | 56 (48.3%)  average dose: 2.1mg/day |
| **Sirolimus** | 5 (4.3%) |
| **Everolimus** | 3 (2.6%) |
| **Donor characteristics:** |  |
| **Male gender** | 76 (65.5%) |
| **Age** [years] | 48 (27 – 57) |
| **BMI** [kg/m^2^] | 24.6 (21.7 – 26.3) |
| **Hypertension** | 36 (31%) |
| **Diabetes** | 7 (6%) |

**S1 Table**
