# Supplementary material for: Serum miR-33a is associated with steatosis and inflammation in patients with non-alcoholic fatty liver disease after liver transplantation
Source: PLoS One. 2019 Nov 8;14(11):e0224820. doi: 10.1371/journal.pone.0224820 (PMC6839850; doi:10.1371/journal.pone.0224820)
Supplement: S2 Table — Data are given as N (%) or median (1st - 3rd quartile). (DOCX) [file pone.0224820.s002.docx]

|  | **Non-steatosis**  **N = 55 (47.4%)** | **Steatosis**  **N = 61 (52.6%)** | **p-value** |
| --- | --- | --- | --- |
| **Immunosupresion:** |  |  |  |
| **Tacrolimus** | 48 (87.3%) | 56 (91.8%) | 0.55 |
| **Cyclosporine** | 7 (12.7%) | 7 (6.6%) | 0.35 |
| **Mycophenolate mofetil** | 33 (60%) | 40 (65.6%) | 0.57 |
| **Azathioprine** | 1 (1.8%) | 1 (1.6%) | 1.0 |
| **Corticosteroids**  [average dose mg/day] | 2.5 (0 – 5) | 0 (0 – 4.5) | 0.26 |
| **Sirolimus** | 2 (3.6%) | 3 (4.9%) | 1.0 |
| **Everolimus** | 1 (1.8%) | 2 (3.3%) | 1.0 |
| **Donor characteristics:** |  |  |  |
| **Male gender** | 24 (43.6%) | 16 (26.2%) | 0.05 |
| **Age** [years] | 46 (27 – 59) | 49 (28 – 56) | 0.64 |
| **BMI** [kg/m^2^] | **23.9 (21.5 – 26)** | **24.9 (23 – 27.8)** | **0.028** |
| **Hypertension** | 18 (32.7%) | 18 (29.5%) | 0.84 |
| **Diabetes** | 4 (7.3%) | 3 (4.9%) | 0.71 |

**S2 Table**
