# Supplementary material for: Serum miR-33a is associated with steatosis and inflammation in patients with non-alcoholic fatty liver disease after liver transplantation
Source: PLoS One. 2019 Nov 8;14(11):e0224820. doi: 10.1371/journal.pone.0224820 (PMC6839850; doi:10.1371/journal.pone.0224820)
Supplement: S3 Table — Data are given as N (%) or median (1st - 3rd quartile). (DOCX) [file pone.0224820.s003.docx]

|  | **Non- inflammation**  **N = 70 (60.3%)** | **Lobular inflammation**  **N = 46 (39.7%)** | **p-value** |
| --- | --- | --- | --- |
| **Immunosupresion:** |  |  |  |
| **Tacrolimus** | 60 (85.7%) | 44 (95.7%) | 0.12 |
| **Cyclosporine** | 9 (12.9%) | 2 (4.4%) | 0.20 |
| **Mycophenolate mofetil** | 40 (57.1%) | 33 (71.7%) | 0.12 |
| **Azathioprine** | 1 (1.4%) | 1 (2.2%) | 1.0 |
| **Corticosteroids**  [average dose mg/day] | 2.5 (0 – 5) | 0 (0 – 2.9) | 0.33 |
| **Sirolimus** | 0 (0%) | 5 (7.1%) | 0.16 |
| **Everolimus** | 1 (1.4%) | 2 (4.4%) | 0.56 |
| **Donor characteristics:** |  |  |  |
| **Male gender** | **30 (43%)** | **10 (22%)** | **0.028** |
| **Age** [years] | 47.5 (24 – 59) | 49 (32.8 – 56) | 0.64 |
| **BMI** [kg/m^2^] | 23.9 (21.5 – 26) | 25 (22.6 – 27.8) | 0.06 |
| **Hypertension** | 22 (31.4%) | 14 (30.4%) | 1.0 |
| **Diabetes** | 5 (7.1%) | 2 (4.4%) | 0.70 |

**S3 Table**
