# Supplementary figures and images for: Serum miR-33a is associated with steatosis and inflammation in patients with non-alcoholic fatty liver disease after liver transplantation
Source: PLoS One. 2019 Nov 8;14(11):e0224820. doi: 10.1371/journal.pone.0224820 (PMC6839850; doi:10.1371/journal.pone.0224820)

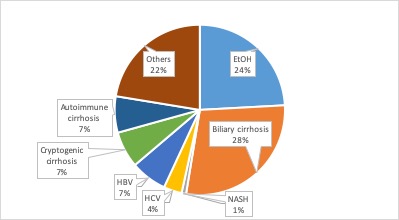

Supplement: S1 Fig — Biliary cirrhosis was indication for LTx in 33 patients (28.5%), alcoholic liver disease in 28 (24.1%), HBV, autoimmune and cryptogenic in 8 patients each (7%), HCV in 4 patients (3.4%), NASH in 1 patient (0.9%) and other diagnoses in 26 patients (22.4%). (JPG) [file pone.0224820.s004.jpg]
